# The Unequal Impact of Disasters: Assessing the Interplay Between Social Vulnerability, Public Assistance, Flood Insurance, and Migration in the U.S

**DOI:** 10.1007/s44212-024-00061-9

**Published:** 2024-11-01

**Authors:** Yu Han, Xinyue Ye, Chunwu Zhu

**Affiliations:** 1 Faculty of Geo-Information Science and Earth Observation (ITC), University of Twente, Enschede, Netherlands; 2 Department of Landscape Architecture and Urban Planning and Center for Geospatial Sciences, Applications and Technology, Texas A and M University, College Station, TX 77840 USA

**Keywords:** Flood Damage, Hazard Mitigation Assistance, Social Vulnerability, Migration, Structure Equation Model

## Abstract

Extreme weather events, such as hurricanes with intense rainfall and storm surges, are posing increasing challenges to local communities worldwide. These hazards not only result in substantial property damage but also lead to significant population displacement. Federal disaster assistance programs are crucial for providing financial support for disaster response and recovery, but the allocation of these resources often unequal due to the complex interplay of environmental, social, and institutional factors. Relying on datasets collected from diverse sources, this study employs a structural equation model to explore the complex relationships between disaster damage (DD), social vulnerability (SV), public disaster assistance (PDA), the national flood insurance (NFI), and population migration (PM) across counties in the contiguous US. Our findings reveal that communities with lower SV tend to experience higher levels of DD across US counties. SV is negatively associated with PM, PDA, and NFI, both directly and indirectly. Furthermore, PDA is positively linked to PM, whereas DD has a direct negative effect on PM but an indirect positive effect through PDA.

## Introduction

Extreme weather and climate-related events, such as hurricanes accompanied by intense rainfall and storm surges, are increasingly challenging community development in the US. The National Oceanic and Atmospheric Administration (NOAA) estimates that damages from such disasters could exceed $100 billion in 2024 alone (NOAA NCEI, 2024). According to Baade et al. (2007), the extensive destruction caused by these severe weather events not only demolishes homes and businesses but also necessitates prolonged periods for rebuilding and recovery. A critical consequence following these disasters is the massive displacement of populations, which serves as key indicators of a community's ability to recover from natural disasters (Ton et al., 2024).

Disaster damage plays a crucial role in shaping migration decisions. The extent of physical destruction can compel residents to leave their homes, especially when the damage is extensive, and recovery appears daunting. For instance, research on Hurricane Katrina reveals that significant housing damage led to both short-term displacement and long-term migration patterns (Fussell et al., 2023). DeWaard et al. (2016) highlight that impediments such as housing damage can cause migration and hinder population recovery in disaster-affected areas, as evidenced in New Orleans after Hurricane Katrina. Similarly, Yabe et al. (2019) found that while many individuals return to their homes shortly after a disaster, a notable portion opts for permanent relocation, influenced by the recovery of infrastructure and housing conditions. Consequently, a deeper understanding of migration trends following disaster damages is essential to enhance disaster risk mitigation planning and improve the efficacy of hazard relief resource allocation.

The challenges presented by the extreme weather and climate disasters would even be amplified for socially vulnerable populations, exacerbated by climate change factors such as sea level rise and more frequent and intensified hurricanes (Dvir et al., 2024; Highfield et al., 2014; Tate et al., 2021). Social vulnerability refers to the capacity of individuals or communities to be adversely affected by external stresses, including natural disasters, economic crises, or social disruptions (Cutter, 2024). Social vulnerability significantly affects communities' ability to handle natural disasters (Cutter et al., 2003; Wang et al., 2019). For example, Finch et al. (2010) highlighted how disparities in vulnerability among New Orleans residents significantly impacted their recovery from Hurricane Katrina, with more vulnerable populations facing greater challenges and being more likely to migrate. Similarly, after Hurricane Ian, the destruction of massive affordable mobile homes along the Florida coast disproportionately affected lower-income residents (Wang et al., 2024). Social stratification can lead to differential recovery rates, where marginalized communities may be forced to relocate due to inadequate support and resources to rebuild (Fussell, 2015). The interplay between social vulnerability, disaster damage, and local risk mitigation policies often results in an unequal distribution of resources that does not strictly align with the level of disaster damage (Waters et al., 2024). As a result, communities with greater social vulnerability—those with fewer resources and less capacity to recover—might receive less disaster recovery assistance than less vulnerable areas, despite potentially suffering greater or equivalent damage (Drakes et al., 2021; Emrich et al., 2022).

Public disaster assistance and risk mitigation programs offer essential financial aid to individuals, businesses, and local governments for effective response and recovery from disasters. Nevertheless, the availability and effectiveness of public disaster assistance can either mitigate or exacerbate migration trends. For example, existing study shows that communities with robust disaster assistance programs tend to experience lower rates of out-migration (West et al., 2013). Conversely, inadequate public disaster assistance can lead to increased out-migration. The reliance on governmental support for recovery may cultivate a dependency, where populations expect public assistance instead of investing in private disaster insurance (Rahim et al., 2023). Flood insurance serves as a critical mechanism in managing the financial risks associated with flooding. Studies indicate that communities with higher levels of flood insurance coverage tend to recover more quickly after disasters, as insured properties can be replaced or repaired without imposing significant financial burdens on the government (Roslan et al., 2019). Tesselaar et al. (2023) argue that the presence of flood insurance can drive population growth in flood-prone areas, as the perceived financial safety encourages settlement despite inherent risks. This relationship suggests that effective flood insurance systems can mitigate the adverse effects of flooding and potentially stabilize or even increase local populations in the aftermath of disasters.

The interplay between social vulnerability, disaster damage, public disaster assistance, and flood insurance creates complex dynamics that significantly influence population migration in communities, which has been less explored in previous research. To investigate these relationships, this study meticulously processed extensive datasets from multiple sources across the US spanning 2001 to 2019 and developed a structure equation model (SEM) to address the following research questions: (1) How do social vulnerability, disaster damage, public disaster assistance, and national flood insurance interact with each other? (2) How do social vulnerability, disaster damage, public disaster assistance, and national flood insurance affect population migrations?

## Disaster risk reduction and recovery in the US

Disaster risk reduction and recovery in the US are primarily managed through a suite of federal programs administered by the Federal Emergency Management Agency (FEMA). Over the past three decades, FEMA has allocated more than $347 billion toward hazard mitigation (Brown, 2015). Key programs include the Hazard Mitigation Assistance (HMA) programs, National Flood Insurance Program (NFIP), Public Assistance (PA) programs, and the Small Business Administration’s (SBA) disaster loan programs. These initiatives play critical roles in enhancing community resilience by addressing disaster risk reduction and facilitating recovery efforts. However, these programs often struggle to effectively serving communities of varying sizes and vulnerabilities.

The HMA serves as a key federal initiative aiming help communities to respond to and recover from future disasters. It provides funding for both post-disaster recovery efforts and proactive pre-disaster projects. Research demonstrates that communities engaging in pre-disaster mitigation strategies are better positioned to withstand and recover from disasters, thereby mitigating socioeconomic impacts (Cox & Hamlen, 2015).

The PA is FEMA's largest financial aid programs providing funds to complement HMA by offering federal assistance for debris removal, emergency protective measures, and the repair or replacement of public infrastructure post-disaster (Davlasheridze & Miao, 2019). The effectiveness of PA is often enhanced when communities have established strong local social networks and capital, which facilitate quicker recovery and adaptation to post-disaster conditions (Pfefferbaum et al., 2017).

The SBA disaster loan programs was established in 1953 to provide a fixed long-term loans with subsidized interest rates for homeowners, renters, and businesses affected by declared natural disasters (SBA, 2021). These loans are particularly crucial for small businesses, providing support to recover and maintain operations when flood insurance and other sources fall short. Studies indicate that SBA loans significantly contribute to community recovery by enabling businesses to access capital, sustain operations, retain employees, and support the local economy(Craig, 2019). However, the distribution of SBA loans often reflects community vulnerabilities, disproportionately affecting socioeconomically disadvantaged areas (Wang & Kang, 2023).

The NFIP provides affordable flood insurance to property owners, renters, and businesses since 1968. It plays a pivotal role in managing flood risks in vulnerable regions. By providing flood insurance accessible and affordable, the NFIP incentivizes property owners to invest in flood mitigation measures, thereby enhancing community resilience against future flood events (Kousky & Kunreuther, 2014; Kousky & Michel-Kerjan, 2017). However, NFIP faces challenges related to affordability and coverage gaps, necessitating reforms to ensure that vulnerable populations can access necessary insurance without excessive financial burden (Kousky & Kunreuther, 2014).

These public programs operate simultaneously in disaster-affected areas, aiming to complement one another. For example, a business might receive flood insurance claims or an SBA loan for immediate repair while applying for an HMA grant for risk mitigation and a PA grant for infrastructure damage repair. Additionally, HMA-funded flood mitigation projects and PA grants could support communities to meet NFIP flood management requirements (Sadiq & Noonan, 2015; Zahran et al., 2010). However, the complexity of these programs poses significant challenges for individuals and governments managing disaster recovery. Each program has different application processes and eligibility criteria, which can be particularly burdensome for vulnerable communities, including low-income and marginalized populations, who may lack the resources to engage these systems effectively (Samuels et al., 2024). Moreover, there are strict rules against "duplication of benefits", preventing individuals or governments receive multiple funds for the same purpose, which can lead to bureaucratic challenges. For example, Kousky et al. (2018) found that increased federal disaster relief often leads to a decrease in average flood insurance coverage, suggesting unintended consequences of overlapping assistance programs.

State governments mandate that local communities, such as counties in Florida, California, and Texas, incorporate risk mitigation planning into their comprehensive plans to minimize natural hazard risks (Lyles et al., 2014). Despite these efforts, challenges persist. For instance, Brody et al. (2013) observed that public disaster assistance grants have not consistently enhanced community resilience, exacerbated by competing demands for resources and post-pandemic economic hardships. Thaler et al. (2019) identified a lack of risk mitigation capacity and insufficient local support as major barriers to implementing effective hazard mitigation strategies. These complexities highlight the urgent need to examine how public disaster assistance is allocated in relation to disaster damage, its influence on population migration, and the extent to which social vulnerability acts as a barrier to the equitable distribution of public disaster assistance and flood insurance. Addressing these issues is crucial for policymakers to design and implement programs that fairly support all communities, particularly the most vulnerable, thereby enhancing overall resilience against future disasters.

## Materials and Methods

### Data collection and processing

The dataset in this research was compiled from multiple sources and further summarized at the County level across the contiguous US. Table 1 listed all datasets and their respective sources. To measure county-level population migration, we utilized data produced by the Internal Revenue Service (IRS) Statistics of Income (SOI) Division. The dataset provides detailed information on the movement of people across different geographical areas within the contiguous US. We aggregated the migration flows to estimate the total annual county-level migration from 2001 to 2019. Positive population migration indicates a population increase in a county over the past three years, and vice versa. We employed the Spatial Hazard Events and Losses Database for the United States (SHELDUS), developed by the Center for Emergency Management and Homeland Security at Arizona State University, to measure the economic losses and impacts of natural hazard events at the county level across the US. The SHELDUS dataset includes information on economic losses and casualties across a wide range of natural hazard events since 1960.

**Table 1:** Description of the Dataset

Data	Description	Sources
Population migration data	County-level population migration estimated based on changes in taxpayer addresses reported to the IRS	IRS Population Migration Data (https://www.irs.gov/statistics/soi-tax-stats-migration-data)
Natural Disaster Damages	County-level natural hazard dataset for the U.S from SHELDUS	Spatial Hazard Events and Losses Database for the United States (SHELDUS) (https://cemhs.asu.edu/sheldus)
National flood insurance dataset	FEMA individual flood insurance policy	FEMA's Data (https://www.fema.gov/about/openfema/data-sets)
Hazard Mitigation Assistance (HMA) dataset	FEMA's Hazard Mitigation program is for mitigating community disaster risk, including both pre-disaster and post-disaster rant programs	
Housing Assistance Program Dataset	FEMA's housing assistance program with disaster declaration for property owners and renters, respectively	
Small Business Assistance (SBA) Dataset	SBA program is to provide low-interest loans for homeowners, home renters, and businesses affected by declared disaster events	SBA Open Data (https://data.sba.gov/)
American Community Survey (ACS)	ACS 5-years county-level community social demographical information in 2019	ACS 5-Years County Level Census Data (https://www.census.gov/geographies/mapping-files/time-series/geo/tiger-data.html)

Datasets on the National Flood Insurance (NFI), Hazard Mitigation, and Housing Assistance (HA) were obtained from FEMA's Data website. FEMA flood insurance contains up-to-date national flood insurance policy information for millions of anonymous individual policyholders from 2001 to 2022. Each flood insurance policy record includes details on the policy count, insurance cost, coverage, CRS class, and county information. The HMA datasets encompass all federal supported hazard mitigation projects since 1989. Each HMA project is linked to a disaster number and includes information such as project category, number of properties, project cost, and county location. The post-disaster programs within the HMA take the majority of HMA projects, which include multiple programs, such as Hazard Mitigation Grants Program (HMGP), Flood Mitigation Assistance (FMA), the Repetitive Flood Claims (RFC) and Severe Repetitive Loss (SRL) programs. In this study, we focused on post-disaster programs as a form of public disaster assistance from the HMA.

The Housing Assistance (HA) dataset is a kind of PA programs that covers individual assistance for all disaster events between 2000 and 2022. This dataset includes public housing assistance for property owners and renters, respectively. The SBA loan datasets from 2001–2019 were collected from SBA open data. They contain information on low-interest disaster loans provided to private homeowners whose properties were damaged by disaster between 2000 and 2019. These datasets provide extensive information to measure hazard exposure, vulnerability, and risk mitigation behaviors in a community. We calculated the total number of projects, the total amount of granted funding, and total number of retrofitted buildings in these projects to assess national flood insurance and public disaster assistance. Additionally, we used the 5-Year County-level census data from the American Community Survey (ACS) in 2019 on social and economic characteristics to measure community social vulnerability. All datasets were aggregated into panel data by summing totals every three years, which served as inputs for the model.

### Structure Equation Model (SEM)

The SEM is designed in this study to measure complex interactions and effects between variables from multiple data sources. Built upon statistical common factor models and path analysis, SEM allows for examining interrelated questions by modeling relationship between multiple dependent and independent variables, as well as observed and latent constructs simultaneously (Kline, 2016). A SEM assumes an underlying model structure that can generate covariances from the observations (Kim, 2003). It is mainly used for estimating multiple interrelated dependence relationships, measuring unobserved obstructs, and explaining complex intercorrelations (Kumar & Kumar, 2015).

The SEM includes two main model components: measurement model and structure model. The measurement model measures the relationships between latent variable and their observed indicators, while the structure model focuses on the dependencies and causal paths among the latent variables. The SEM models have the strengths of simultaneously performing a confirmatory factor analysis (CFA) and path analysis. The CFA in the SEM shows relationship between laten variables and their indicators. Path analysis test hypothesized relationships among latent and observed variables.

In the measurement model, there are coefficients that represent the relationship between each latent variable and its indicators, which is called factor loadings. High factor loadings indicate that the indicators are good measures of the latent construct. In the structure model, there are endogenous variables and exogenous variables. The endogenous variables are dependent latent variables that are influenced by other variables in the model. The exogenous variables are independent latent variables that influence other variables but are not influenced by any other variables within the model. The standardized coefficients between two variables in the structure model that indicate the strength and direction of the relationships between latent variables are called path coefficients. SEM has the advantage to measure both direct and indirect effects between exogenous variables and endogenous variables (SOBEL, 1987). The direct effects are the effects of one variable directly on another within the structural model, and the indirect effects are the effects of one variable on another that are mediated through one or more intervening variables.

**Fig. 1:**
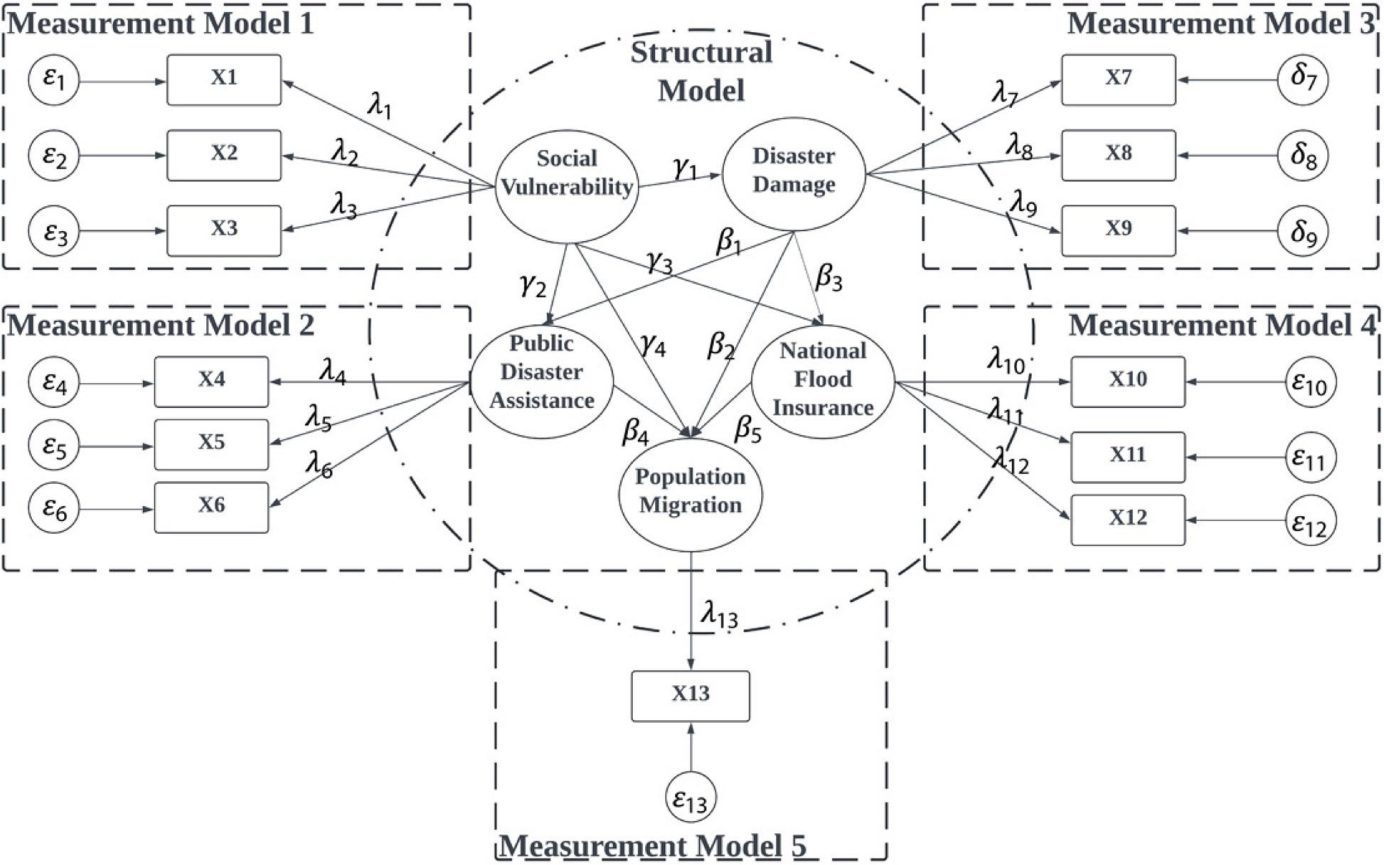
An Illustration of the Structure Equation Model (SEM) in this Study with Structure and Measurement Components

To build the SEM model, five latent variables were selected to represent Social Vulnerability (SV), Disaster Damage (DD), Public Disaster Assistance (PDA), National Flood Insurance (NFI), and Population Migration (PM). Figure 1 illustrated the developed SEM with structure and measurement components. We identified the SEM model structure through trails and error measurements. The structure model indicates the complex interactions between latent variables. There are two kinds of path coefficients in the structure mode, namely $\gamma$ and $\beta$. The $\gamma$ represents path coefficients from exogenous variables to endogenous variables, and the $\beta$ indicates path coefficients from endogenous variables to endogenous variables. There are five measurement models in Fig. 1. In each measurement model, each selected indicator has a residual. The $\varepsilon$ represents residuals of y-side indicators, and the $\delta$ represents residuals on the x-side indicators. The factor loading ($\lambda$) represent the relationship between observed indicators and their underlying latent variable. Many tools available for building SEM. The Lavaan package in R has been widely used for SEM studies. It has the advantages of user-friendly syntax, comprehensive capabilities, flexibility, and robust statistical methods. Therefore, it was used for building and analyzing SEM in this study.

### Processing model variables

We first standardized all variables and then applied a collinearity diagnostic approach to remove variables with high variance inflation factors (VIF). We then chose observed indicators based on statistical criteria of the built model. Finally, a total of 14 variables are selected to measure the 5 latent variables, and their statistical summaries are shown in Table 2. Population migration is measured as the total net population migration. A positive value represents population growth in that period, and vice versa. Four variables were chosen to measure social vulnerability in a county. They are the percentage of elderly population, the percentage of unemployed population, and the percentage of population below poverty. We did not include social demographic variables, such as the percentage of minorities and the average household income, as they either reduced model performance or had negative coefficients in the measurement model. For disaster damage, we adopted total amount of property damage and crop damage, as well as total number of fatalities in a period. To measure public disaster assistance, we choose the total HA for homeowners, the total post-disaster HMA, and the total SBA. The HA for homeowners measures total amount of housing assistance for homeowners every three years between 2001 and 2019. The total post-disaster HMA represents total amount of funding for post-disaster hazard mitigation projects in each time period. Similarly, the total small business loans are adopted to measure total investments of post-disaster small business loan in a time period. To measure national flood insurance policy, we chosen the total flood insurance policy count, the average cost of flood insurance policy, and the total amount of building coverage in a county.

**Table 2:** Variable Description and Summary Statistics

Category	Variables	Description	Mean	St. dev	Min	Max
Population Migration (PM)	IRS Net Population Change	Net population migration at the county level, estimated based on changes in taxpayer addresses reported to the IRS	0.78	2822	-77,692	1.36E + 05
Disaster Damage (DD)	Property Damage	Total property damage at the county level due to flood-related disasters from SHELD, presented on a log scale	7.13	6.39	0.00	23.79
	Crop Damage	Total crop damage at the county level from the SHELD, presented on a log scale	1.44	4.07	0.00	20.03
	Fatality	Total fatality at the county level from the SHELD	0.18	4.07	0.00	638
Social Vulnerability (SV)	Percent of Elderly Population	The percentage of the population aged 65 and older in a county	0.19	0.05	0.03	0.56
	Percent of Unemployed Population	The percentage of the unemployed population in a county	0.74	0.04	0.53	0.90
	Percent of Population below Poverty	The percentage of the population that is unemployed in a county	0.14	0.04	0.04	0.33
Public Disaster Assistance (PDA)	Total HA for Homeowners	Total amount of Housing Assistance (HA) for homeowners, presented on a log scale	1.14	3.56	0.00	19.59
	Total Post-Disaster Assistance	Total amount of post-disaster risk mitigation assistance, presented on a log scale	1.78	4.44	0.00	20.19
	Total SBA loans	Total amount of Small Business Assistance (SBA) loans, presented on a log scale	1.74	4.34	0.00	20.27
National Flood Insurance (NFI)	Flood insurance policy count	The number of flood insurance policies in a county	965.60	7803.58	0	3.39E + 05
	Average flood insurance cost	The average flood insurance cost in a county	607.5	631.4857	0	18,002
	Total Flood Insurance Coverage	Total flood insurance coverage for all buildings in a county, presented on a log scale	7.231	5.814586	0	15.202

## Results

### Structure model results

We evaluate model results based on multiple model performance metrics. The scaled root means square error (RMSEA), the comparative fit index (CFI), and Tucker–Lewis index (TLI) are used to evaluate model results. RMSEA is absolute fit indexes to explain how far the fitted model is from the perfect model. An RMSEA or SRMR < 0.06 indicates a good fit, and < 0.08 suggests a reasonable model–data fit. For CFI and TLI, a value larger than 0.9 indicates a good model fit (Xia & Yang, 2019; Zhu et al., 2024). Our final model result has the RMSEA 0.051. The CFI is 0.936 and TLI is 0.912, which indicate that the fitted model could well explain hidden model structure in the dataset.

A total of 14 observed indicators were used to measure five laten variables. As show in Table 3, three observed indicators construct Social Vulnerability. The percentage of elderly population has the highest factor loading, while the percentage of the unemployed population has the lowest factor loading. For the three observed indicators for disaster damage, property damage contributes most significantly. For public disaster assistance, total SBA and total HA for homeowners both have higher factor loadings than total post-disaster assistance. This indicates that individual level assistances have more contribution on the latent variable. For national flood insurance, total flood insurance coverage has the highest factor loading. This shows the level of flood insurance coverage in a community.

**Table 3:** Latent variables from the SEM model fitting

Latent Variable	Observed Variable	Factor Loading	Std	*P*-value
SV	Percent of the elderly population	1.156	0.014	***
	Percent of the unemployed population	0.385	0.007	***
	Percent of the population below poverty	0.415	0.006	***
DD	Crop damage	0.305	0.004	***
	Property damage	1.073	0.009	
	Fatality	0.053	0.010	***
PDA	Total HA for homeowners	0.786	0.010	***
	Total post-disaster assistance	0.380	0.007	***
	Total small business assistance (SBA) loans	0.835	0.008	***
NFI	Flood Insurance Policy Count	0.104	0.004	***
	Average Flood Insurance Cost	0.742	0.009	
	Total Flood Insurance Coverage	1.038	0.013	***
PM	IRS Net Population Change	0.998	0.048	***

Significance condition *P*-value: < 0.001 ‘***’ < 0.01 ‘**’ < 0.05 ‘*’ < 0.1 ‘.’

### Measurement model results

**Fig. 2:**
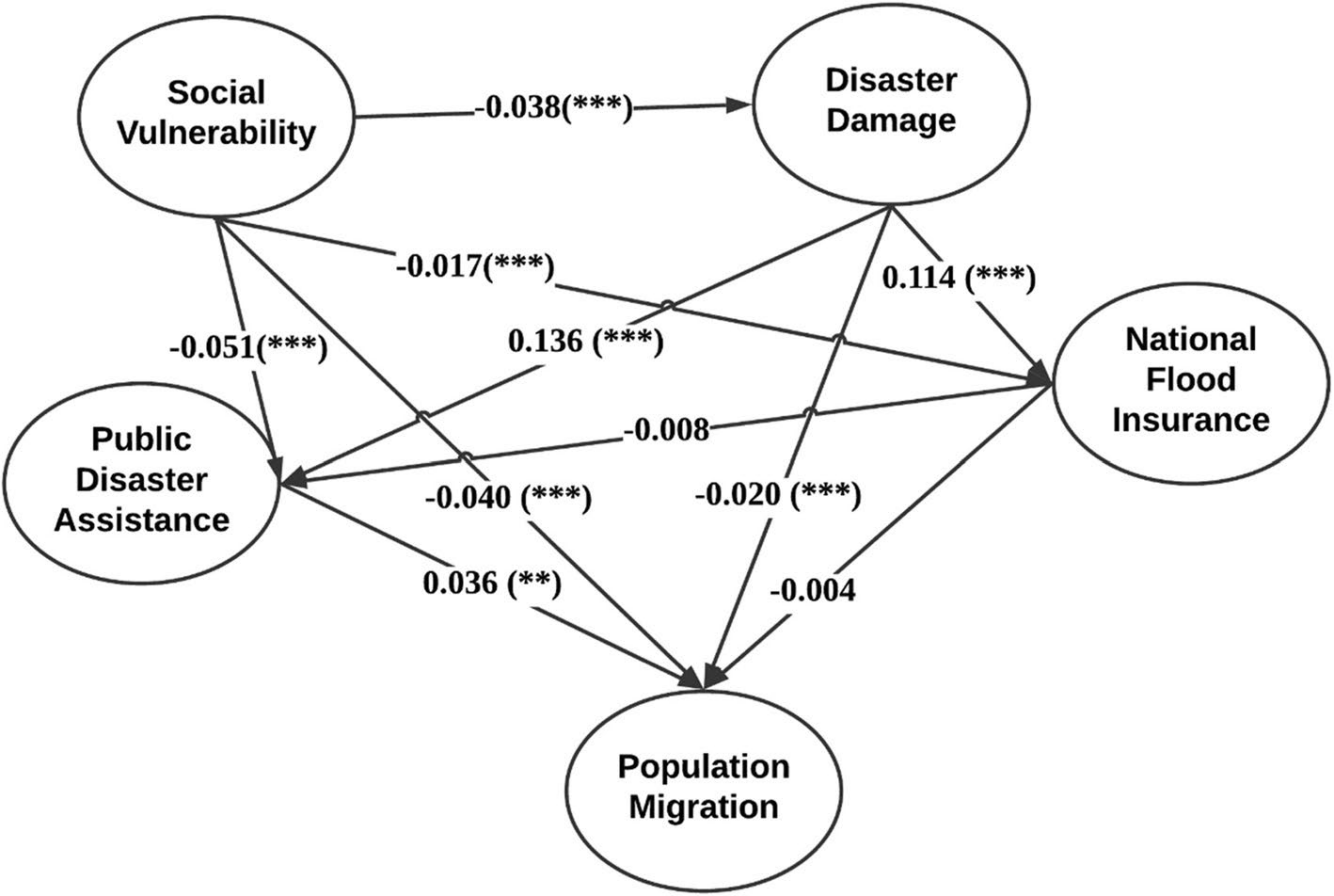
The Structural Model Displaying Standardized Path Coefficients Among Social Vulnerability (SV), Disaster Damage (DD), Public Disaster Assistance (PDA), National Flood Insurance (NFI), and Population Migration (PM)

Figure 2 displays the fitted model graph and path coefficients, illustrating the complex relationships between latent variables. The path analysis reveals that all path coefficients, except for the National Flood Insurance, are statistically significant. Given that all observed indicators are standardized to have a mean of zero and a standard deviation of one, the results indicate the expected changes in the dependent variable (in standard deviations) for one standard deviation change in the predictor.

The coefficient of PDA to PM is 0.036 at a 0.001 significant level. This means one standard deviation increase in PDA is associated with a 0.036 standard deviations increase in PM. Conversely, both SV and DD negatively affect PM, indicating that higher levels of these factors might lead to decline in community populations. The coefficient of SV to PM is -0.040 at a 0.001 significant level and the coefficient of DD to PM is -0.020 at a 0.001 significant level. These results mean one standard deviation increase in SV and one standard deviation increase in DD is associated with a 0.040 standard deviation and 0.020 standard deviation decrease in PM, respectively. SV is negatively linked with DD, with -0.038 coefficient at a 0.001 significant level. This result implies that communities with higher social vulnerability tend to experience lower levels of disaster damages. This counterintuitive result suggests a complex relationship between SV and DD at the county level, which may counteract the coexistence situation of social vulnerability and disaster damages within communities.

Furthermore, DD positively associate with both PDA and NFI at a 0.001 significant level, indicating that communities experiencing greater disaster damages also receive more public assistance and are more likely to have higher flood insurance coverage. One standard deviation increase in DD is associated with 0.136 standard deviation increase in PDA and 0.114 standard deviation increase in NFI. However, SV has an opposite effect: The coefficients of SV to PDA and NFI are -0.051 and -0.017 at a 0.001 significant level, respectively. This means one standard deviation increase in SV is associated with 0.051 standard deviation decreases in PDA and 0.017 standard decreases in NFI.

NFI negatively linked with both PDA and PM. The coefficients of NFI to PDA and PM are -0.008 and -0.004, respectively. This reveals higher flood insurance coverage might reduce public disaster assistance and would lead to population decrease. Since the public disaster assistance is mainly represented by individual level disaster assistance, including housing assistance and low interest loans, this result suggests that while flood insurance may provide financial security against disasters, it might reduce the need for household level public disaster assistance in communities. Additionally, an increase in flood insurance coverage is associated with deterred population growth in counties, suggesting that while insurance may provide financial security against disasters, it could also discourage new or sustained residency. Overall, these findings illustrate the complex interplay between social factors and disaster management measures.

### Direct and indirect model effects

SEM can estimate both direct effects and indirect effects among variables. As shown in Table 4, both direct and indirect paths are observed among the latent variables. Specifically, the latent variables, SV and DD, have indirect effects on PM. The indirect coefficient of SV on PM through PDA is -1.83e-3, accounting for 4.23% of the total effects, while the indirect effect of SV on PM through DD is 7.6e-4, constituting 1.79% of the total effects based on absolute values. The indirect effect of DD on PM through PDA is 4.9e-3, which represent 19.32% of the total effects based on absolute values. These results illustrate that while PDA partially mediates the relationship between DD and PM, the direct negative effect of DD on PM remains predominant. Moreover, the direction of indirect effects of DD on PM through PDA is opposite to its direct effect, a phenomenon known as "competitive mediation". This implies that while disaster damage is generally associate with population decrease in a county, areas that suffer higher disaster damage but receive high public disaster assistance would have less population decrease. For example, more affluent coastal communities may experience population growth given their high exposure to natural hazards. This illustrates how natural disasters damages influence population migration through multiple opposing pathways. To mitigate the negative effects of natural disasters on community population migration, effective interventions could focus on enhancing public disaster assistance. The indirect effects of SV on PDA and NFI through DD are -5.2e-3 and -4.3e-3, respectively. These negative indirect effects of SV on PDA and NFI align with the direction of their corresponding direct effects. This indicates that DD mediates the relationship between SV and PDA, as well as between SV and NFI, contributing to 9.25% and 20.18% of their total effects, respectively.

**Table 4:** Direct and indirect effects of model results from the SEM model

Latent Variables	Regression Path	Effect Type	Coefficient	Percentage to the total effect
PM	SV—> PM	Direct Effect	-0.04	93.98%
	PDA—> PM	Total/Direct Effect	0.036	100.00%
	DD—> PM	Direct Effect	-0.02	78.86%
	NFI—> PM	Total/Direct Effect	-4.0e-3	100.00%
	DD- > PDA- > PM	Indirect Effect	4.9e-3	19.32%
	DD- > NFI- > PM	Indirect Effect	-4.6e-4	1.81%
	SV- > PDA- > PM	Indirect Effect	-1.8e-3	4.23%
	SV- > DD- > PM	Indirect Effect	7.6e-4	1.79%
PDA	SV—> PDA	Total/Direct Effect	-0.051	90.74%
	DD—> PDA	Total/Direct Effect	0.136	100.00%
	SV—> DD—> PDA	Total/Direct Effect	-5.2e-3	9.25%
	NFI—> PDA	Total/Direct Effect	-0.011	100.00%
NFI	SV—> NFI	Direct Effect	-0.017	79.81%
	SV- > DD- > NFI	Indirect Effect	-4.3e-3	20.18%
	DD—> NFI	Total/Direct Effect	0.114	100.00%
DD	SV—> DD	Total/Direct Effect	-0.038	100.00%

## Conclusions

This study integrated multiple sources of datasets to investigate the dynamics among social vulnerability, natural hazard damages, public disaster assistance, and flood insurance and their impacts on migration at the county level. Results identified complex relationships among latent variables SV, DD, PDA, and NFI, each affecting PM and disaster management efforts in different ways. SV and DD both exert a negative influence on migration. High levels of either factor is associated with a decline in community populations, indicating that more socially vulnerable or heavily damaged areas are less attractive for residents. Nevertheless, DD is positively linked with PM through PDA. This result implies that the strong intermediate effects of public assistances in disaster risk management. SV is negatively associated with NFI through DD, which also indicates a significant portion of the relationship between SV and NFI is explained by DD. PDA positively associates with PM, suggesting that effective post-disaster support can reduce population out-migration in affected communities. DD exhibits positive correlations with PDA and NFI, suggesting areas with more severe damages receive more support and are more likely to have better insurance coverage.

These findings indicate the complex interplay of factors affecting disaster management and their varied impacts on population dynamics. The results highlight the crucial role of effective disaster assistance and flood insurance in supporting community resilience and recovery. However, the negative impacts of social vulnerability on migration and public resource allocation reveal the needs for policy interventions in natural disaster risk reduction. Results indeed suggest a need for enhanced public disaster assistance and insurance coverage at the federal level to increase the resilience of socially vulnerable communities to natural disasters.

The data-driven approach adopted in this study clear indicated competitive mediation with disaster management, which have been underexplored in previous research. It shows how DD can both negatively affect PM through direct path and positively affect PM through indirect paths, as well as its negative association with SV and positive association with PDA and NFI. Previous studies focused on isolated impacts of disasters on the migration of socially vulnerable population or the equitable distribution of post-disaster assistance (Griego et al., 2020; Myers et al., 2008). This research integrates multiple factors into a SEM to improve our understanding on the relationships among social vulnerability, natural disaster, public disaster assistance, national flood insurance, and migration in the US. The findings elucidate the complex interrelationships among these factors and competing effects that these factors have on population migration in the US over the past two decades.

This study also has some shortcomings that could be improved in future studies. First, this study was conducted on the county level and could not reflect variations within communities. For example, existing studies have found that high levels of disaster impacts often coexist with high social vulnerability (Flores et al., 2020). However, results of this study found a negative association between disaster damage and social vulnerability on the county level across the US. Secondly, this study utilized 2019 ACS County data to measure social vulnerability at the county level. Future research could use census data from different time periods to examine dynamic changes in social vulnerability and its association with disaster damage, public disaster assistance, and population migration. Thirdly, the SEM developed in this study could not effectively capture the temporal effects of disaster impacts, public assistance, and social factors. Therefore, future studies could consider incorporating time-varying relationships of social vulnerability, public disaster assistance, and population migration for individual disaster events to better understand these complex interactions over time.

## Data Availability

The data that support the findings of this study are available from the corresponding author upon reasonable request.
